# White matter microstructures in Parkinson's disease with and without impulse control behaviors

**DOI:** 10.1002/acn3.51504

**Published:** 2022-02-09

**Authors:** Haruka Takeshige‐Amano, Taku Hatano, Koji Kamagata, Christina Andica, Wataru Uchida, Masahiro Abe, Takashi Ogawa, Yasushi Shimo, Genko Oyama, Atsushi Umemura, Masanobu Ito, Masaaki Hori, Shigeki Aoki, Nobutaka Hattori

**Affiliations:** ^1^ Department of Neurology Juntendo University Faculty of Medicine 2‐1‐1 Hongo, Bunkyo‐ku Tokyo 1138421 Japan; ^2^ Department of Neurology Juntendo University Nerima Hospital 3‐1‐10 Takanodai Nerima‐ku Tokyo 1778521 Japan; ^3^ Department of Radiology Juntendo University Faculty of Medicine 2‐1‐1 Hongo, Bunkyo‐ku Tokyo 1138421 Japan; ^4^ Department of Neurosurgery Juntendo University Faculty of Medicine 2‐1‐1 Hongo, Bunkyo‐ku Tokyo 1138421 Japan; ^5^ Department of Psychiatry Juntendo University Faculty of Medicine 2‐1‐1 Hongo, Bunkyo‐ku Tokyo 1138421 Japan; ^6^ Department of Radiology Toho University Omori Medical Center 6‐11‐1 Omorinishi, Ota‐ku Tokyo 1438540 Japan

## Abstract

**Background:**

Impulse control behaviors (ICBs) in Parkinson's disease (PD) are thought to be caused by an overdose of dopaminergic therapy in the relatively spared ventral striatum, or by hypersensitivity of this region to dopamine. Alterations in brain networks are now also thought to contribute to the development of ICBs.

**Objective:**

To comprehensively assess white matter microstructures in PD patients with ICBs using advanced diffusion MRI and magnetization transfer saturation (MT‐sat) imaging.

**Methods:**

This study included 19 PD patients with ICBs (PD‐ICBs), 18 PD patients without ICBs (PD‐nICBs), and 20 healthy controls (HCs). Indices of diffusion tensor imaging (DTI), diffusion kurtosis imaging, neurite orientation dispersion and density imaging, and MT‐sat imaging were evaluated using tract‐based spatial statistics (TBSS), regions of interest (ROIs), and tract‐specific analysis (TSA).

**Results:**

Compared with HCs, PD‐nICBs had significant alterations in many major white matter tracts in most parameters. In contrast, PD‐ICBs had only partial changes in several parameters. Compared with PD‐ICBs, TBSS, ROI, and TSA analyses revealed that PD‐nICBs had lower axial kurtosis, myelin volume fraction, and orientation dispersion index in the uncinate fasciculus and external capsule, as well as in the retrolenticular part of the internal capsule. These are components of the reward system and the visual and emotional perception areas, respectively.

**Interpretation:**

Myelin and axonal changes in fibers related to the reward system and visual emotional recognition might be more prominent in PD‐nICBs than in PD‐ICBs.

## Introduction

Impulse control disorders (ICDs), including gambling disorders, compulsive shopping, compulsive sexual behaviors, and binge eating, occur in around 8%–40% of Parkinson's disease (PD) patients.^1^, ^2^, ^3^ Hobbyism/punding and dopamine dysregulation syndrome are classified as ICD‐related behaviors, and their underlying pathologies are thought to be similar to those of ICDs. These behaviors are considered impulsive control behaviors (ICBs).^4^ ICBs are thought to be caused by an overdose of dopaminergic therapy in the relatively spared ventral striatum, or by dopamine denervation‐induced hypersensitivity of D3 receptors in the ventral striatum.^5^ Moreover, recent investigations have reported that alterations not only of the dopaminergic system, but also of brain networks might contribute to the development of ICBs in PD.^6^

Diffusion‐weighted magnetic resonance imaging (DW‐MRI) techniques have been developed to be able to detect intracerebral microstructural changes in PD.^7^, ^8^, ^9^, ^10^, ^11^, ^12^, ^13^ However, conventional diffusion tensor imaging (DTI) is not suitable for non‐Gaussian diffusion, which is often the case in biological tissues. In addition, the interpretation of DTI parameter estimations is often difficult because DTI parameters reflect various microstructural changes. Multi‐shell DW‐MRI modalities, including diffusion kurtosis imaging (DKI) and neurite orientation dispersion and density imaging (NODDI), can overcome this weakness. DKI reflects the non‐Gaussianity of distribution,^7^ and NODDI captures the density and orientation dispersion of neurites,^13^ enabling a more detailed analysis of various structures. Indeed, both of these multi‐shell DW‐MRI techniques have been previously reported to be more sensitive to cerebral microstructural changes in PD than conventional DTI.^7^, ^9^, ^11^ Furthermore, magnetization transfer saturation (MT‐sat) imaging can be used to estimate the myelin volume fraction (MVF).^14^ MT saturation (MT‐sat) imaging was developed to improve magnetization transfer ratio imaging (MTR), a classic myelin measurement, by decoupling MTR from R1.^15^ Compared with MTR, MT‐sat has been reported to better correlate with disability severity in multiple sclerosis patients.^16^

One conventional DTI study revealed that several areas, including orbitofrontal areas, the corpus callosum, and the internal capsule, are preserved in PD patients with ICDs compared with PD patients without ICDs.^17^ Additionally, a study investigating structural connectivity revealed the relative preservation of the connection between the substantial nigra and the mesolimbic system.^18^ However, there have been no detailed assessments of white matter microstructures in PD patients with ICBs; thus, the precise white matter alterations associated with ICB in PD remain unclear. On the basis of these findings, we evaluated white matter microstructures using advanced DW‐MRI and myelin imaging techniques to elucidate the pathological white matter pathways of ICBs in PD.

## Methods

### Participants

We used brain MRI to prospectively evaluate PD patients with ICBs (PD‐ICB), PD patients without ICBs (PD‐nICB), and healthy controls (HCs). All groups were approximately matched for age and sex. Patients with major abnormalities in the standard MRI, or those diagnosed with dementia with PD or dementia with Lewy bodies according to the Movement Disorders Society (MDS) criteria, were excluded.^10^, ^19^ PD was diagnosed according to the MDS Clinical Diagnostic Criteria for PD.^20^ The clinical conditions of PD patients were evaluated with Hoehn and Yahr (H&Y) stages and the MDS‐Unified Parkinson's Disease Rating Scale (MDS‐UPDRS) parts I and III.^21^ Patients who scored one point or more in the Questionnaire for Impulsive‐compulsive Disorders, Japanese edition (QUIP‐J) were diagnosed as PD‐ICB.^22^ The levodopa equivalent daily dose (LEDD) was calculated according to a previous systematic review.^23^

### Ethics statement

The study protocol complied with the Declaration of Helsinki and was approved by the ethics committee of Juntendo University (14–011). Written informed consent was given by all participants.

### MRI acquisition

All participants were scanned on a 3‐T MRI scanner (MAGNETOM Prisma, Siemens Healthcare, Erlangen, Germany) using a 64‐channel head coil. Diffusion weighted imaging (DWI) was obtained using spin‐echo planar imaging consisting of two *b* values of 1000 and 2000 s/mm^2^, completed with a *b* = 0 image with no diffusion gradients. PD patients were scanned during the “on” state. DWI data were acquired along 64 isotropic diffusion gradients in the anterior–posterior phase‐encoding direction. Standard and reverse phase‐encoded blipped images with no diffusion weighting (blip up and blip down) were acquired to correct for magnetic susceptibility‐induced distortions related to the echo‐planar imaging acquisitions.^24^, ^25^, ^26^ The sequence parameters were as follows: repetition time, 3300 ms; echo time, 70 ms; field of view, 229 × 229 mm; matrix size, 130 × 130; resolution 1.8 × 1.8 mm; slice thickness, 1.6 mm; and acquisition time, 7 min 29 s.

The 3D multi‐echo fast low‐angle shot sequences were performed with predominant T1‐, proton density‐, and MT‐weighting for calculating the MT‐sat index. Acquisition parameters for MT‐sat were as follows: MT‐off and MT‐on scanning = repetition time, 24 ms; echo time, 2.53 ms; flip angle, 5°; and T1‐weighted imaging = repetition time, 10 ms; echo time, 2.53 ms; flip angle, 13°; parallel imaging using GeneRalized Autocalibrating Partial Parallel Acquisition factor 2 in the phase‐encoding direction; 7/8 partial Fourier acquisition in the partition direction; bandwidth, 260 Hz/pixel; field of view, 224 × 224 mm; matrix size, 128 × 128; slice thickness, 1.8 mm; and acquisition time, 6 min 25 s.

### MRI preprocessing

All DWI datasets were corrected for susceptibility‐induced geometric distortions, eddy current distortions, and inter‐volume subject motion using EDDY and TOPUP toolboxes.^25^ The resulting images were fitted to the NODDI model^27^ using NODDI MATLAB Toolbox 5 (http://www.nitrc.org/projects/noddi_toolbox). Maps of intracellular volume fraction (ICVF) and orientation dispersion index (ODI) were acquired using accelerated microstructure imaging via convex optimization. The diffusion‐weighted data were then processed using the Diffusional Kurtosis Estimator^28^ implemented in MATLAB (Math‐Works, Natick, MA, USA). Maps of mean kurtosis (MK), axial kurtosis (AK), and radial kurtosis (RK) were generated. The diffusion tensor was estimated using ordinary least squares applied to the DWI, with *b* = 0 and 1000 s/mm^2^. Maps of fractional anisotropy (FA) and mean diffusivity (MD) were calculated using the Diffusion Toolbox tool implemented in the FMRIB Software Library (FSL; Oxford Centre for Functional MRI of the Brain, Oxford, UK), based on standard formulae.^29^

MT‐sat data were used to calculate the MVF using an in‐house MATLAB script based on the theory described previously.^30^ Furthermore, to create MVF maps, the MT‐sat index was calibrated with a factor of 0.1.

### Tract‐based spatial statistics analysis

Voxel‐wise statistical analysis was carried out using tract‐based spatial statistics (TBSS), which is part of FSL^26^ version 5.0.9. This analysis was performed to regionally map significant differences between groups in each of the DTI, DKI, NODDI, and MT‐sat indices using the skeleton projection step. First, all the subjects' FA maps were aligned to a common Montreal Neurological Institute 152 space using the FMRIB Nonlinear Registration Tool.^26^ Second, the mean FA image was created and thinned to create the mean FA skeleton, which represented the centers of all tracts common to the groups. The mean FA skeleton was then thresholded to FA > 0.20, to include the major white matter pathways and exclude peripheral tracts and gray matter. Third, the aligned FA map of each subject was projected onto the FA skeleton. Finally, similar steps were performed for the DTI (MD), DKI (MK, RK, and AK), NODDI (ICVF and ODI), and MT‐sat (MVF) maps, such that without the initial registration, these maps were projected onto the mean FA skeleton.

### Region of interest analysis

Any maps that showed significant clusters in the TBSS analyses were localized using the John Hopkins University ICBM‐DTI‐81 white matter labels and white matter tractography atlases.^31^, ^32^ We first extracted five areas each for MD and AK, three areas for ODI, and seven areas for MVF that had significant differences between PD‐ICBs and PD‐nICBs in the TBSS analyses, and then evaluated the regions of interest (ROIs) of these areas. Post hoc analyses were performed on the ROIs that survived multiple comparison adjustments and those that were chosen based on previous reports.^17^, ^33^

### Tract‐specific analysis

We performed tract‐specific analysis (TSA) to validate the ROI analyses using the probabilistic Multi‐Shell Multi‐Tissue Constrained Spherical Deconvolution (MSMT‐CSD)^34^ tracking method with the iFOD2 algorithm, implemented in the MRtrix 3.0.0 software package (Brain Research Institute, Melbourne, Australia, http://www.brain.org.au/software/). We estimated five white matter tracts in which significant differences were observed between PD‐ICBs and PD‐nICBs in the ROI analysis. The pathways were categorized in the ROI analysis (Table S1) using the following parameters: step size = 1.0 mm; maximum curvature = 45° per step; length = 4–200 mm; and fiber orientation distribution threshold = 0.05. All tractography from seed to target ROIs included a total of 50 × 10^4^ streamlines for each fiber tract. The FreeSurfer^35^ version 6.0.0 pipeline was used to define the ROIs located in cortical gray matter according to Desikan‐Killiany cortical atlas segmentation.^36^ In addition, subcortical gray matter ROIs were obtained from subcortical gray matter partial volume fraction maps for more accurate segmentation using the FMRIB Integrated Registration and Segmentation Tool in the FSL. The mean AK, MVF, and ODI were then measured for each pathway bilaterally using the “tcksample” script implemented in the MRtrix 3.0 software.

### Statistical analysis

All statistical analyses were performed using JMP 13 (SAS Institute Inc., Addison, TX, USA) and GraphPad Prism 8 (GraphPad Software, Inc., San Diego, CA, USA), except for the general linear model (GLM) analyses, which were performed using FSL, and the chi‐squared test, which was performed using js‐STAR version 9.7.0j (http://www.kisnet.or.jp/nappa/software/star/freq/chisq_ixj.htm#).

**Figure 1:**
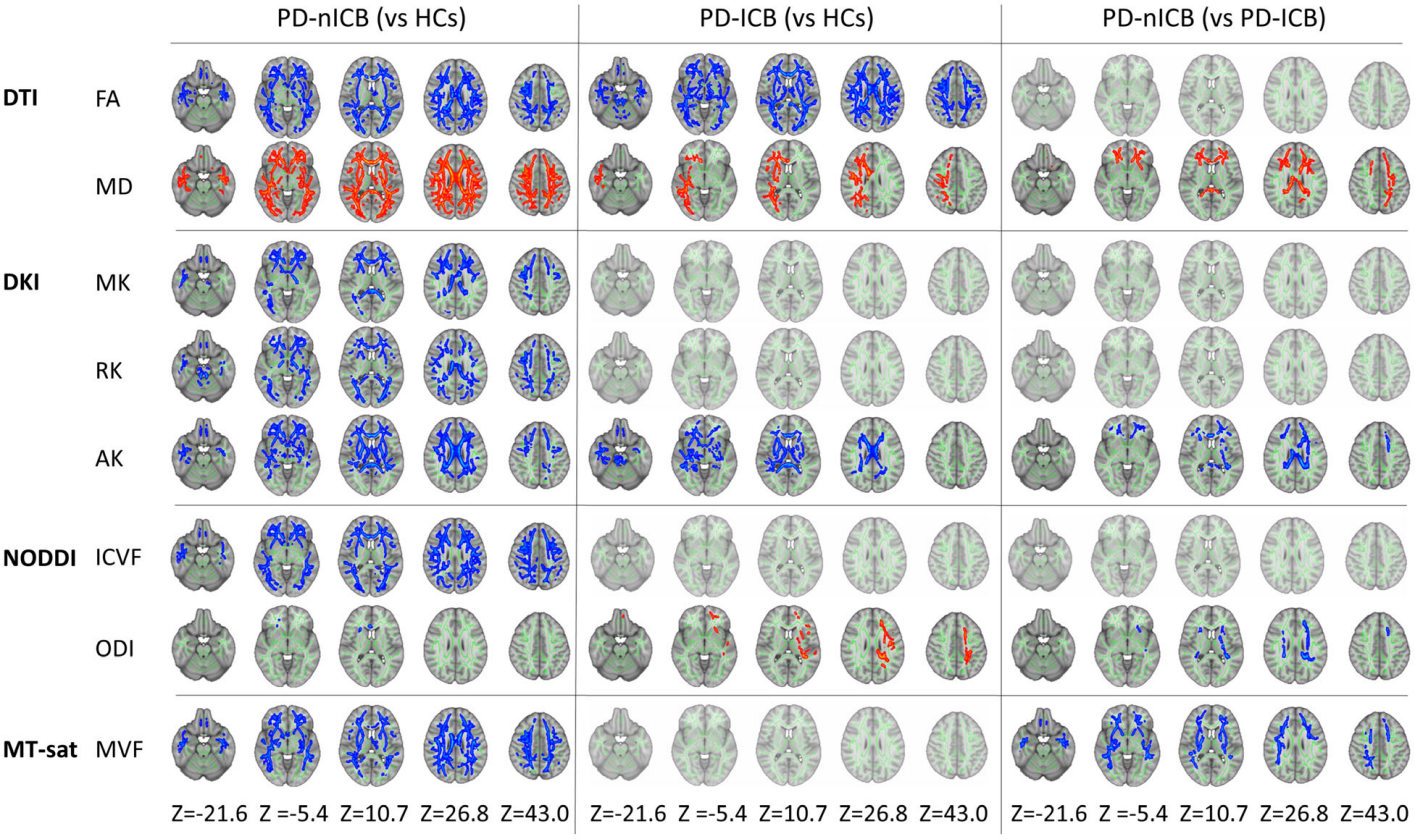
Significant areas in each parameter detected by TBSS analysis. The significant areas in each parameter are shown. Areas in red had significantly higher values of each index in the comparison between the two groups, while those in blue had lower values. AK, axial kurtosis; FA, fractional anisotropy; ICVF, intracellular volume fraction; MD, mean diffusivity; MK, mean kurtosis; MVF, myelin volume fraction; ODI, orientation dispersion index; PD‐ICBs, Parkinson's disease patients with impulse control disorders; PD‐nICBs, Parkinson's disease patients without impulse control disorders; RK, radial kurtosis; TBSS, tract‐based spatial statistics. [Colour figure can be viewed at wileyonlinelibrary.com]

For TBSS analyses, a voxel‐wise GLM framework with one‐way analysis of variance, which included age and sex (Fig. 1) with LEDD (Fig. 5) as covariates, was applied to compare all diffusion metrics between groups using the FSL randomize tool with 5000 permutations. Results were then corrected for multiple comparisons by controlling family‐wise error (FWE, which is most commonly used in TBSS) for the number of voxels and applying threshold‐free cluster enhancement. A *p*FWE of less than 0.05 was considered significant.

For the ROI analyses and TSA, we used the Kruskal–Wallis test for nonparametric parameters and one‐way analysis of variance for parametric parameters. False discovery rate controlling procedures were then applied to correct for multiple testing. For post hoc analyses, we used the Mann–Whitney *U* test for nonparametric parameters and Student's *t*‐test for parametric parameters, to compare each pair out of the three groups. One‐way analysis of covariance (ANCOVA) was performed using LEDD as a covariate to evaluate the effect of LEDD. Spearman's rank correlation test was used for the correlation analysis between LEDD and each parameter. A *p*‐value of less than 0.05 was considered statistically significant.

## Results

### Participants

Table 1 shows the participant characteristics of each group, including age, sex, disease duration, H&Y stages, MDS‐UPDRS part I and III scores, QUIP‐J scores, LEDD, and LEDD of dopamine agonists (LEDD‐DA). We recruited 20 participants for each group; however, one PD‐nICB patient was excluded because he had a microtubule‐associated protein tau mutation. We also excluded patients with disease durations of less than 5 years because disease duration affects the prevalence of ICBs. Thus, we finally enrolled 19 participants in the PD‐ICB group, 18 in the PD‐nICB group, and 20 in the HC group. Total LEDD and LEDD‐DA were both significantly higher in PD‐ICBs (*p* = 0.0320 and 0.0159, respectively), as expected. There were no significant differences in disease duration, H&Y stages, and MDS‐UPDRS part I and III scores between the PD‐ICB and PD‐nICB groups. The symptoms of ICBs are shown in the “QUIP” row in Table 1. Most patients had more than two symptoms.

**Table 1:** Participant characteristics.

	HCs N = 20	PD‐ICB N = 19	PD‐nICB N = 18	*p*‐value
Age	66.75 ± 1.07	67.11 ± 7.00	66.28 ± 5.03	0.734
Gender (M/F)	9/11	10/9	5/13	n.s.^a^
Disease duration	NA	14.3 ± 7.75	10.2 ± 4.82	0.103
Hoehn & Yahr	NA	3.05 ± 0.97	2.20 ± 0.924	0.102
UPDRS‐III	NA	23.79 ± 16.29	23.4 ± 12.13	0.903
UPDRS‐I	NA	9.63 ± 5.56	8.11 ± 3.60	0.473
QUIP (number of patients)	NA	2.21 ± 2.27 Pathological gambling (1), Hypersexuality (4), Compulsive shopping (8), Compulsive eating (6), Punding (11), DDS (7)	0	NA
LEDD	NA	1320.58 ± 936.55	852.72 ± 398.87	0.0320
LEDD‐DA	NA	242.10 ± 165.36	112.92 ± 134.09	0.0159

HCs, healthy controls; PD‐ICBs, Parkinson's disease patients with impulse control behaviors; PD‐nICBs, Parkinson's disease patients without impulse control behaviors; UPDRS, Movement Disorders Society‐Unified Parkinson's Disease Rating Scale; QUIP, Questionnaire for Impulsive‐compulsive Disorders; DDS, dopamine dysregulation syndrome; LEDD, levodopa equivalent daily dose; DA, levodopa equivalent daily dopamine agonist dose; NA, not applicable.

^a^ *p*‐value was obtained using the chi‐squared test; other *p*‐values were obtained using the Kruskal–Wallis test.

### Tract‐based spatial statistics analysis

In TBSS, there were apparent diffuse changes in the white matter of PD‐nICBs compared with HCs, such as higher MD and lower MK, RK, AK, ICVF, and MVF. In contrast, areas with significant differences were restricted to a partial elevation of MD and reduction of AK in the PD‐ICBs compared with HCs (Fig. 1). The significant areas were distributed broadly to the thalamic radiation, corona radiata, sagittal stratum, internal and external capsules, superior and inferior fronto‐occipital fasciculus, superior and inferior longitudinal fasciculus, uncinate fasciculus, cingulum cingulate gyrus, corticospinal tract, cerebellar peduncle, forceps major and minor, and corpus callosum (Table S1). In contrast, the ODI value in the PD‐ICB group was significantly higher (more abnormal) than in the PD‐nICB and HC groups. Compared with PD‐nICBs, PD‐ICBs had significantly lower MD and higher AK and MVF (more preserved) in tracts, including the corpus callosum, internal and external capsules, corona radiata, thalamic radiation, cingulum, superior and inferior longitudinal fasciculus, inferior fronto‐occipital fasciculus, and uncinate fasciculus. PD‐ICBs also had higher ODI (more abnormal), mainly in the left superior longitudinal fasciculus, corona radiata, and internal capsule (Fig. 1). FA was significantly lower in both PD‐ICBs and PD‐nICBs compared with HCs, but there were no significant differences between the two PD groups.

### Region of interest analysis

Significant differences in the MD, AK, ODI, and MVF of certain areas were found between the PD‐ICB and PD‐nICB groups in the TBSS analysis; thus, the ROIs of these values were evaluated. ROIs from left white matter structures were evaluated for ODI analysis because changes were almost always localized to the left side. For the analysis of other indices, the mean values of bilateral structures were evaluated. On the basis of the TBSS results, five areas each for MD and AK, three areas for ODI, and seven areas for MVF were analyzed. Among these areas, one area for MD, three areas for AK, one area for ODI, and three areas for MVF were significant. Of these areas, the splenium of the corpus callosum (SCC) for AK and the external capsule for MVF survived the multiple comparison adjustments (Table S1). On the basis of these results and previously reported pathological areas associated with ICBs and PD, further analysis was performed on five areas.^17^, ^33^

**Figure 2:**
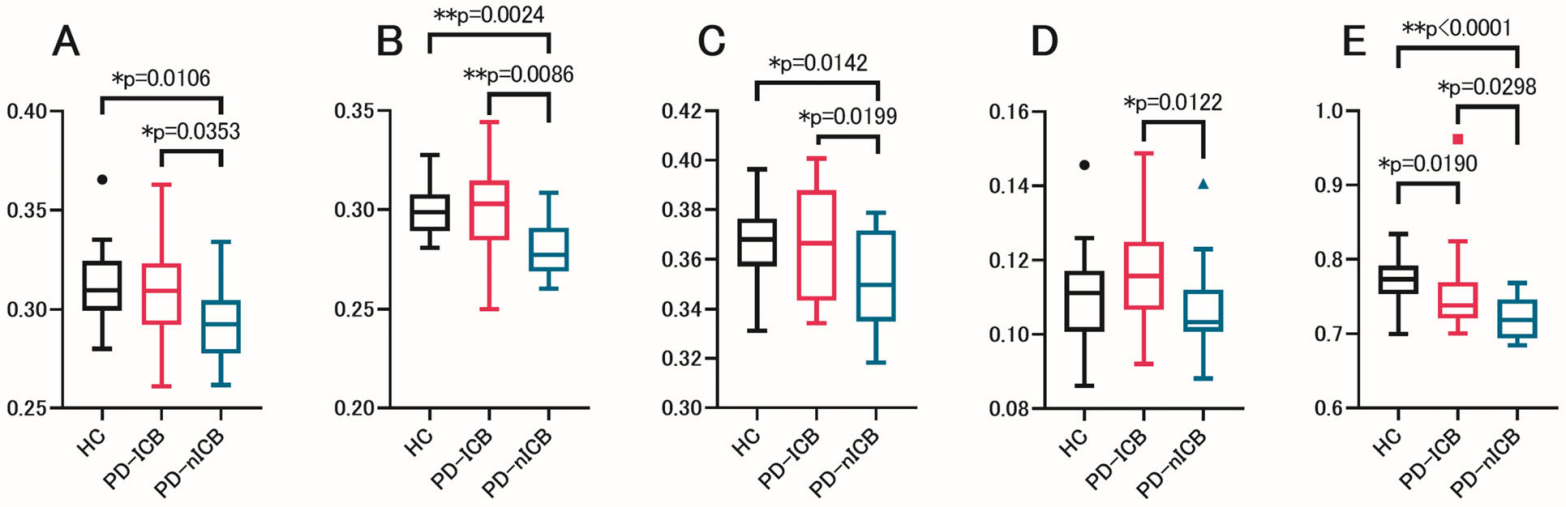
ROI analysis revealed five white matter tracts significantly associated with ICBs. The mean values and standard errors of parameters with significantly different areas between PD‐ICBs and PD‐nICBs are shown. (A). MVF in the uncinate fasciculus. (B). MVF in the external capsule. (C). MVF in the ILF. (D). ODI in the RLIC. (E). AK in the SCC. AK, axial kurtosis; HC, healthy controls; ILF, inferior longitudinal fasciculus; MVF, myelin volume fraction; ODI, orientation dispersion index; PD‐ICBs, Parkinson's disease patients with impulse control disorders; PD‐nICBs, Parkinson's disease patients without impulse control disorders; RLIC, retrolenticular part of the internal capsule; ROI, region of interest; SCC, splenium of the corpus callosum. *p*‐values were obtained using the Mann–Whitney test. [Colour figure can be viewed at wileyonlinelibrary.com]

Compared with the PD‐ICBs and HCs, the PD‐nICBs showed notable alterations in most parameters, including a significantly lower AK (more abnormal) in the SCC (HCs: *p* < 0.0001, PD‐ICBs: *p* = 0.0298) and lower MVFs (more abnormal) in the inferior longitudinal fasciculus (ILF; HCs: *p* = 0.0142, PD‐ICBs: *p* = 0.0199), uncinate fasciculus (HCs: *p* = 0.0106, PD‐ICBs: *p* = 0.0353), and external capsule (HCs: *p* = 0.0024, PD‐ICBs: *p* = 0.0086) (Table S1, Fig. 2). No differences were detected between the PD‐ICBs and HCs in these areas except for the AK of the SCC (*p* = 0.0190). Only the ODI value was abnormal in the PD‐ICBs, with a significantly higher ODI in the retrolenticular part of the internal capsule (RLIC) compared with the PD‐nICBs (*p* = 0.0122); there was no difference between the PD‐nICBs and HCs.

### Tract‐specific analysis

**Figure 3:**
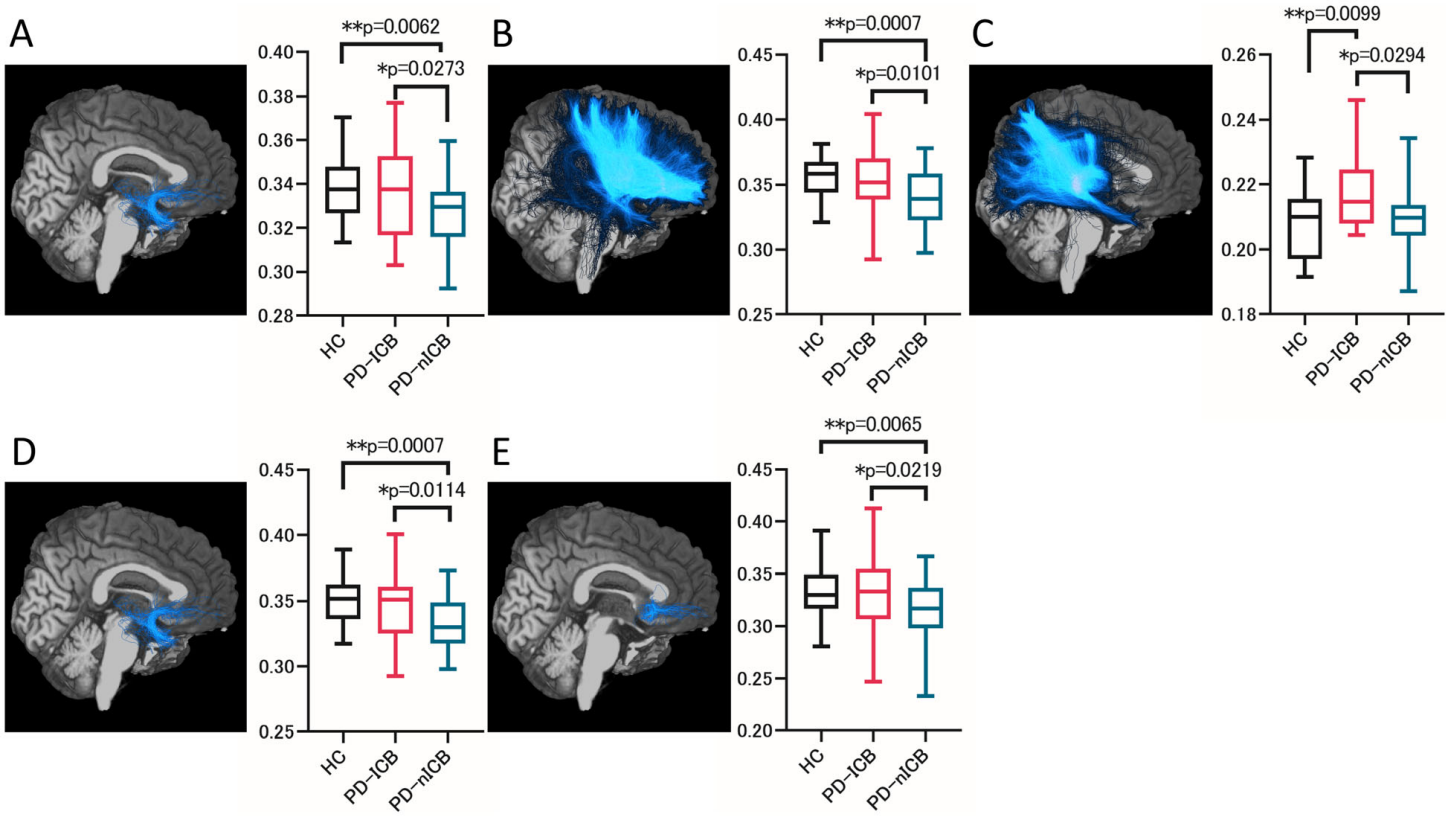
Tractography of significant pathways and tract‐specific analysis of each parameter. Tractography of the significant pathways is shown with the tract‐specific analysis results of each parameter. (A). Uncinate fasciculus and MVF of this tract. (B). External capsule and MVF of this tract. (C). RLIC and ODI of this tract. (D). Salience network and MVF of this tract. (E). Pathway from the lateral orbitofrontal area to the accumbens area and MVF of this tract. AK, axial kurtosis; HC, healthy controls; MVF, myelin volume fraction; ODI, orientation dispersion index; PD‐ICBs, Parkinson's disease patients with impulse control disorders; PD‐nICBs, Parkinson's disease patients without impulse control disorders; RLIC, retrolenticular part of the internal capsule. *p*‐values were obtained using the Mann–Whitney *U* test and Student's *t*‐test. [Colour figure can be viewed at wileyonlinelibrary.com]

**Figure 4:**
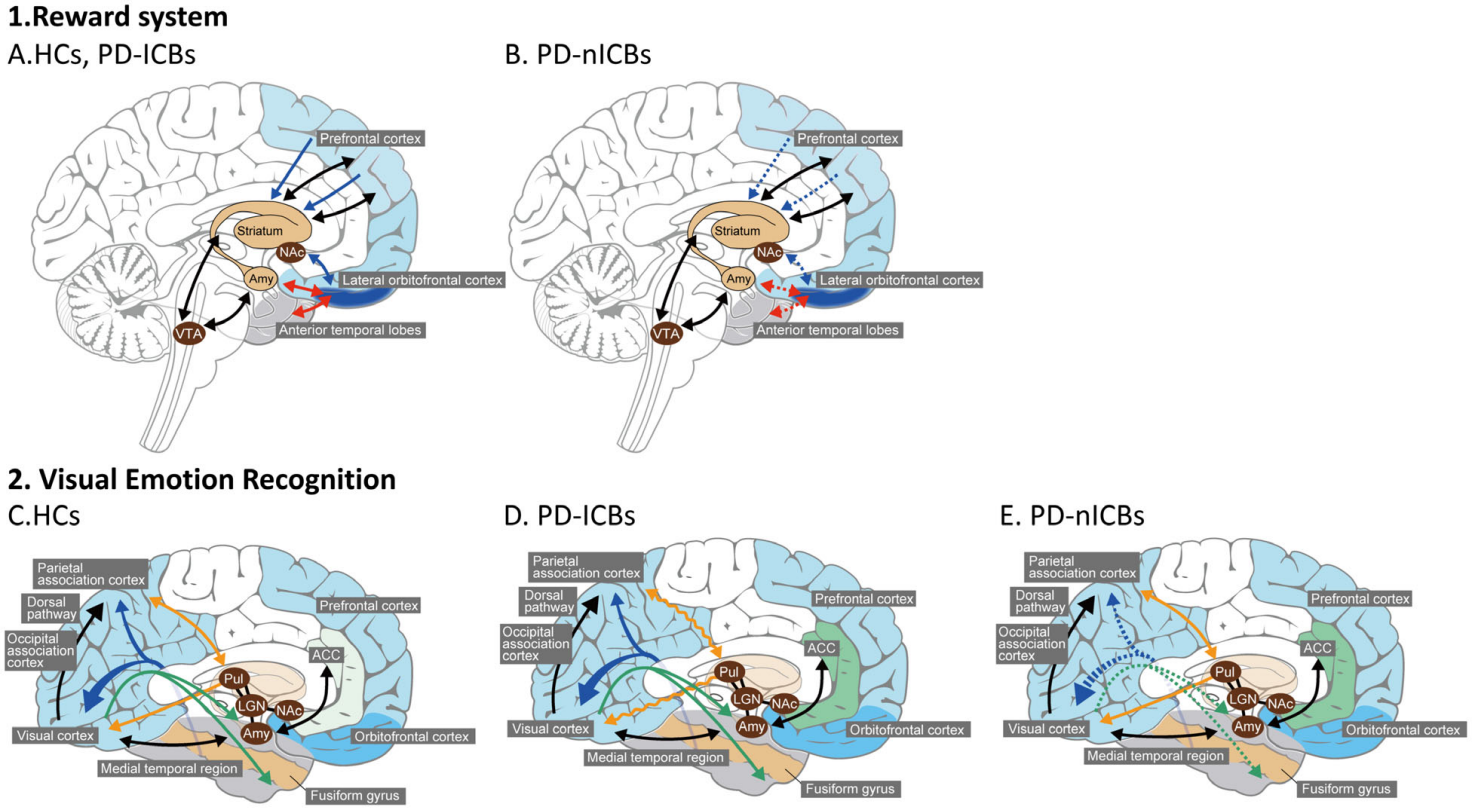
Schemas of significant pathways. Brain networks involved in the reward system (A, B) and visual emotion recognition (C, D, E) are shown. The reward system in HCs and PD‐ICBs (A), and in PD‐nICBs (B). The MVF of the external capsule (blue arrows) and the uncinate fasciculus (red arrows) was significantly decreased in PD‐nICBs compared with that in the other two groups. Visual emotion recognition networks in HCs (C), PD‐ICBs (D), and PD‐nICBs (E). The ODI of the RLIC (yellow arrows) was higher in PD‐ICBs than in HCs and PD‐nICBs. The SCC (blue arrows) and the ILF (green arrows) were altered in PD‐nICBs, with lower AK and MVF, respectively. ACC, anterior cingulate cortex; AK, axial kurtosis; Amy, amygdala; HCs, healthy controls; ILF, inferior longitudinal fasciculus; LGN, lateral geniculate nucleus; NAc, nucleus of accumbens; ODI, orientation dispersion index; PD‐ICBs, Parkinson's disease patients with impulse control disorders; PD‐nICBs, Parkinson's disease patients without impulse control disorders; Pul, pulvinar; RLIC, retrolenticular part of the internal capsule; SCC, splenium of corpus callosum; VTA, ventral tegmental area. [Colour figure can be viewed at wileyonlinelibrary.com]

TSA, which was performed to validate the ROI analyses, revealed almost the same results: significantly lower MVFs in the uncinate fasciculus and external capsule in PD‐nICBs and a significantly higher ODI in the RLIC in PD‐ICBs compared with those in the other groups (Figs. 3 and 4). There were no significant differences in AK in the SCC and MVF of the ILF, but a similar tendency to that of the ROI results was observed. The MVF of the “salience network”, which is one of the large‐scale brain networks, was significantly lower in the PD‐nICBs than in the other two groups. Furthermore, among the pathways from the orbitofrontal cortex, one toward the accumbens area had a significantly lower MVF in the PD‐nICBs, whereas no differences were detected between the PD‐ICB and HC groups.

### Association of each parameter with LEDD

**Figure 5:**
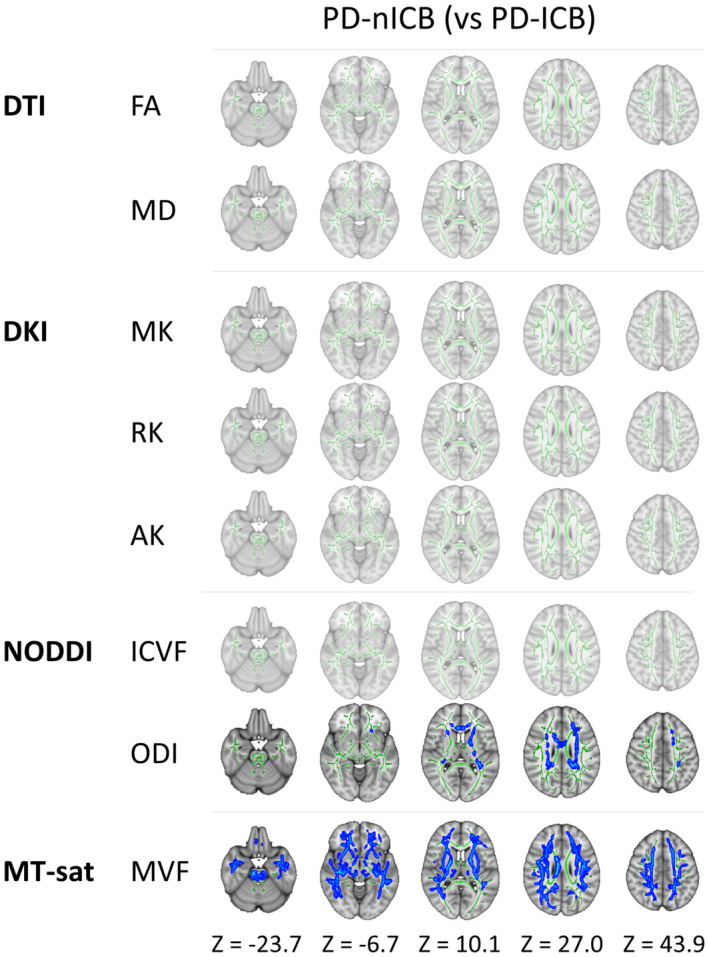
Significantly different areas between PD with and without ICBs, analyzed using age, sex, and LEDD as covariates. TBSS analysis was performed using age, sex, and LEDD as covariates. Areas in red had significantly higher values of each index in the comparison between the two groups, whereas those in blue had lower values. AK, axial kurtosis; FA, fractional anisotropy; ICVF, intracellular volume fraction; MD, mean diffusivity; MK, mean kurtosis; MVF, myelin volume fraction; ODI, orientation dispersion index; PD‐ICBs, Parkinson's disease patients with impulse control disorders; PD‐nICBs, Parkinson's disease patients without impulse control disorders; RK, radial kurtosis; TBSS, tract‐based spatial statistics. [Colour figure can be viewed at wileyonlinelibrary.com]

An additional analysis that included LEDD, age, and sex as covariates (Fig. 5) revealed that there was a significant difference in ODI and MVF between the PD‐ICB and PD‐nICB groups. Among the parameters with significant differences, only ODI of RLIC was positively correlated with LEDD (ρ= 0.329, *p* = 0.0468). To evaluate the effect of LEDD on ODI of RLIC, we performed an ANCOVA using LEDD as a covariate for both the ROI and TSA analyses. A significant difference in ODI‐RLIC was found in the TSA analysis after adjustment (*p* = 0.0335). The ROI analysis revealed the same tendency, although it did not reach significance (*p* = 0.0948).

## Discussion

To elucidate the pathological white matter microstructure changes that occur with ICBs in PD, we comprehensively evaluated cerebral white matter in HCs and in PD patients with and without ICBs using the latest MRI modalities: DTI, DKI, NODDI, and MT‐sat imaging. Table S1 describes the DW‐MRI parameters used in this study and their clinical significance.^37^ To our knowledge, this is the first report of DKI, NODDI, and MT‐sat imaging being used to evaluate white matter microstructure alterations in PD patients with and without ICBs. Marked changes in ICVF, MVF, and indices of DTI and DKI were detected in certain white matter regions in PD‐nICBs, while white matter alterations were relatively restricted in PD‐ICBs. In addition, ODI was significantly elevated in PD‐ICBs compared with PD‐nICBs and HCs. These findings suggest the diffuse abnormality of white matter microstructures in idiopathic PD patients, which has been previously reported.^7^, ^10^

On the basis of our results, we detected five significant areas associated with ICBs: the uncinate fasciculus, external capsule, ILF, RLIC, and SCC.

### Hyperdopaminergic stimulation of the relatively preserved reward system

The uncinate fasciculus and the external capsule are both involved in the reward system.^38^, ^39^ The human reward system includes midbrain dopaminergic neurons, especially in the ventral tegmental area, striatum, amygdala, and orbitofrontal cortex. The uncinate fasciculus connects the anterior temporal lobes to the anterior insula, lateral orbitofrontal cortex, cingulate gyrus, and frontal pole (Fig. 4A,B, red arrows).^39^ The external capsule carries fibers to the striatum from the prefrontal cortex, including the orbitofrontal cortex (Fig. 4A,B, blue arrows).^38^ The MVFs in these areas were significantly lower in PD‐nICBs, suggesting demyelination of these pathways (Fig. 4B). Among these fibers, TSA revealed a significantly lower MVF in the tract from the orbitofrontal cortex to the accumbens area in PD‐nICBs compared with PD‐ICBs and HCs (Figs. 3 and 4B). The uncinate fasciculus is associated with various functions, including visceral and emotional integration, object–reward association learning, and behavioral inhibition,^39^, ^40^ and is thought to overlap with the salience network, which is involved in the rewarding process and interoceptive awareness.^40^ In a resting‐state functional MRI study, connectivity of the salience network was spared in drug‐naïve PD patients who went on to develop ICBs, while in other PD patients it was significantly decreased compared with HCs.^6^ In the present study, TSA revealed that both the salience network and the uncinate fasciculus were more preserved in PD‐ICBs than in PD‐nICBs.

Impulsivity was associated with the relative preservation of mesolimbic connectivity in patients with PD. Consistent with our results, the preservation of connectivity between the substantia nigra and limbic network targets has been reported in PD patients with ICBs.^18^ D3 agonist‐induced hyperdopaminergic stimulation might revitalize the reward system in PD‐ICBs but might not activate the reward system in PD‐nICBs because of degeneration.

### Other significant pathways

The RLIC contains tracts that run from the pulvinar and lateral geniculate nuclei to the association and visual cortices (Fig. 4C–E, yellow arrows). ^41^ The SCC contains anterior fibers that connect medial temporal regions, including the fusiform cortex, to the contralateral parietal associative areas, and posterior fibers to the visual area (Fig. 4C,D,E, blue arrows).^39^, ^42^ ILF is another association tract that carries visual information from occipital areas to the temporal lobe.^39^ These areas, as well as the uncinate fasciculus, are associated with visual and emotional recognition. PD patients have visual emotion recognition impairments, with reduced activity in the striatum, amygdala, orbitofrontal cortex, and temporal facial recognition areas.^43^ Identification of sadness is reported to correlate with FA changes in the ILF.^44^ These areas overlap decision‐making and reward systems; orbitofrontal cortex degeneration in PD patients is associated with the impairment of both decision‐making and facial emotion recognition.^43^ In our study, the mean AK of the SCC was significantly lower in PD‐nICBs than in PD‐ICBs and HCs, indicating chronic axonal damage (Fig. 4E, blue dotted arrows), and MVF of the ILF was significantly lower, indicating demyelination (Fig. 4E, green dotted arrows).^45^ In PD‐ICBs, both the ROI analysis and TSA revealed abnormal ODI in the left RLIC, indicating decreased integrity of the tract (Fig. 4D, yellow wiggly arrows). Visual recognition pathways via the left RLIC are closely associated with ICBs; left amygdala volume is reported to increase in PD‐ICBs.^46^ An overdose of dopaminergic therapy to these preserved systems may induce modifications, detected as ODI elevations, in PD‐ICBs.

Our findings support a previous DTI study^17^ indicating that white matter integrity in PD patients with ICDs is relatively preserved. However, the previous investigation only assessed whole‐brain voxel‐based measures, based on a conventional DTI study. The current study is notable because tracts associated with reward systems in PD‐nICBs showed decreased MVF, suggesting myelin alterations, whereas tracts associated with facial emotion recognition showed changes in AK, ODI, and MVF, suggesting both axonal and myelin alterations. There is no pathological evidence of demyelination in PD patients, but myelin damage has been suggested by DTI studies.^47^ Recent studies have revealed that α‐synuclein (AS) oligomers, which are considered the toxic form, might be more widely distributed than Lewy pathologies.^48^ Additionally, AS oligomers negatively impact axonal transport.^49^ An impairment of white matter microstructures caused by AS oligomers might be reflected in altered DW‐MRI parameters. Furthermore, it is important to note that TSA confirmed the results, supporting our hypotheses regarding the pathways associated with ICBs.

Our study had several limitations. First, PD diagnoses were not determined pathologically, and the number of patients were relatively small. Second, pathological studies are needed to investigate the exact meaning of each MRI parameter in PD patients with and without ICBs. Third, the ICB diagnoses were only based on QUIP‐J scores. However, all cases with ICBs also had specific symptoms clinically confirmed. Although HCs were not assessed for ICBs using QUIP‐J, we recruited HCs without any neurological or psychiatric disorders based on detailed interviews of their medical history. Finally, in our cohort, there was a significant difference in LEDD and LEDD‐DA between the PD‐ICD and PD‐nICD groups. Additionally, the groups were not perfectly sex‐matched, although they were not significantly different. These factors may affect the results. However, the TBSS results were almost the same even when analyzed using age, sex, and LEDD as covariates (Fig. 5 and Table S1). Moreover, it is interesting to note that white matter was less altered in PD‐ICB subjects who received higher doses of medication and had relatively longer disease duration, on average. Nevertheless, further studies are needed to evaluate the effects of antiparkinsonian drugs on white matter microstructures.

In conclusion, DKI and MT‐sat imaging revealed less change in white matter microstructures—especially those associated with the reward system and visual and emotional recognition—in PD‐ICBs than in PD‐nICBs, and NODDI was able to detect a characteristic change in the white matter of PD‐ICBs. PD patients with these preserved pathways may therefore be vulnerable to ICBs.

## Conflict of Interest

The authors have no conflict of interest to report.

## Supporting information


**Table S1** Seed and target ROIs for each pathway analyzed in tract‐specific analysis.
**Table S2.** TBSS analysis of DTI, DKI, NODDI, and MT‐sat indices in PD patients with and without ICB vs. healthy controls, and in PD patients with ICB vs. PD patients without ICB.
**Table S3.** ROI analysis of the indices with significant differences between PD patients with and without ICBs.
**Table S4.** TBSS analysis of ODI and MVF in Parkinson's disease patients with and without impulsive‐compulsive behaviors adjusted for age, sex, and levodopa equivalent daily dose
**Table S5.** Methods and parameters used in this study
