# Supplementary material for: White matter microstructures in Parkinson's disease with and without impulse control behaviors
Source: Ann Clin Transl Neurol. 2022 Feb 9;9(3):253–63. doi: 10.1002/acn3.51504 (PMC8935280; doi:10.1002/acn3.51504)
Supplement: Supplementary file 1 — Table S1 Seed and target ROIs for each pathway analyzed in tract‐specific analysis. Table S2. TBSS analysis of DTI, DKI, NODDI, and MT‐sat indices in PD patients with and without ICB vs. healthy controls, and in PD patients with ICB vs. PD patients without ICB. Table S3. ROI analysis of the indices with significant differences between PD patients with and without ICBs. Table S4. TBSS analysis of ODI and MVF in Parkinson's disease patients with and without impulsive‐compulsive behaviors adjusted for age, sex, and levodopa equivalent daily dose Table S5. Methods and parameters used in this study [file ACN3-9-253-s001.docx]

**Supplementary Table 1. Seed and target ROIs for each pathway analyzed in tract specific analysis.**

| **Pathways** | **Seed ROI** | **Target ROI** |
| --- | --- | --- |
| **Uncinate facsiculus** | insula | Superior temporal |
|  | medial orbitofrontal | inferior temporal |
|  | lateral orbitofrontal | Parahippocampal |
|  |  | Temporal pole |
|  |  | amygdala |
| **Exernal capsule** | caudate | caudal middle frontal |
|  | putamen | rostral middle frontal |
|  |  | lateral orbitofrontal |
|  |  | medial orbito frontal |
|  |  | pars opercularis |
|  |  | pars orbitalis |
|  |  | pars triangularis |
|  |  | precentaral |
|  |  | superior temporal |
|  |  | inferior temporal |
|  |  | lingual |
|  |  | cuneus |
| **Inferior longitudinal fasciculus** | Lateral occipital | Middle temporal |
|  | Lingugal | Inferior temporal |
|  | Fusiform | Temporal pole |
|  | cuneus | Parahippocampal |
|  |  | hippocampus |
|  |  | amygdala |
| **Retrolenticular part of internal capsule** | thalamus | superior parietal lobule |
|  |  | inferior parietal lobule |
|  |  | superior temporal gyrus |
|  |  | lingual |
|  |  | cuneus |
|  |  | pericalcarine |
|  |  | thalamus postarior cingulate |
|  |  | isthmuscingulate |
| **Splenium of corpus callosum** | Lingual | Lingual |
|  | Cuneus | Cuneus |
|  | Superior parietal | Superior parietal |
|  | Parahippocampal | Parahippocampal |
|  | Amygdala | Amygdala |
|  | Fusiform | Fusiform |
|  | Posterior cingulate | Posterior cingulate |
|  | Isthmus cingulate | Isthmus cingulate |
| **Salience network** | Insula | caudal antriror cingulae |
|  |  | rostral anterior cingulate |
|  |  | lateral orbitofronatal |
|  |  | medial orbitofrontal |
| **Lateral orbitofrontal - Accumbents pathway** | accumbents | lateral orbitofrontal |

Abbreviation: ROI, region of interest.

**Supplementary Table 2. TBSS analysis of DTI, DKI, NODDI, and MT-sat indices in PD patients with and without ICB vs. healthy controls, and in PD patients with ICB vs. PD patients without ICB.**

| **Modality** | **Contrast** | **Cluster size** | **Anatomical region** | **Peak**  ***T*-value** | **Peak MNI coordinates**  **(X, Y, Z)** |
| --- | --- | --- | --- | --- | --- |
| **DTI** | | | | | |
| FA | HCs  > PD-nICB | 25644 | Bilateral ATR, CST, CCG, CgH, IFOF, ILF, SLF, UF, SLF temporal part, medial lemniscus, PLIC, retrolenticular part of IC, ACR, SCR, PCR, PTR, sagittal stratum, and external capsule; Lt-ALIC and CP; forceps major and minor, genu, body, and splenium of CC, and fornix. | 6.35 | 121, 166, 72 |
|  | HCs  > PD-ICB | 32166 | Bilateral ATR, CST, CCG, CgH, IFOF, ILF, SLF, UF, SLF temporal part, medial lemniscus, ICP, SCP, CP, ALIC, PLIC, retrolenticular part of IC, ACR, SCR, PCR, PTR, sagittal stratum, external capsule, and SFOF; forceps major and minor, MCP, PCT, genu, body, and splenium of CC, and fornix. | 6.17 | 130, 87, 70 |
| MD | HCs  < PD-nICB | 30989 | Bilateral ATR, CST, CCG, IFOF, ILF, SLF, UF, SLF temporal part, ALIC, PLIC, retrolenticular part of IC, ACR, SCR, PCR, PTR, sagittal stratum, external capsule, and SFOF; Rt-CP; forceps major and minor, genu, body, and splenium of CC, and fornix. | 6.35 | 41, 85, 62 |
|  | HCs  < PD-ICB | 7034 | Rt-ATR, CST, IFOF, ILF, SLF, UF, SLF temporal part, ALIC, PLIC, retrolenticular part of IC, ACR, SCR, PCR, and PTR; forceps major and minor, genu, body, and splenium of CC. | 5.46 | 41, 85, 62 |
|  | PD-nICB  > PD-ICB | 7372 | Bilateral ATR, IFOF, retrolenticular of IC, ACR, SCR, PCR, and PTR; Lt-CST and UF; Rt-SLF, SLF temporal part, ALIC, external capsule and SFOF; forceps major and minor, genu, body, and splenium of CC. | 5.24 | 99, 89, 86 |
| **DKI** | | | | | |
| MK | HCs  > PD-nICB | 17502 | Bilateral ATR, CCG, IFOF, SLF, UF, ALIC, PLIC, ACR, SCR, PCR, PTR, external capsule, and SFOF; Lt-CST, CP, and PLIC; Rt-ILF, SLF temporal part, and retrolenticular part of IC; forceps major and minor, genu, body, and splenium of CC. | 6.09 | 104, 83, 92 |
| RK | HCs  > PD-nICB | 21146 | Bilateral ATR, CST, CCG, IFOF, ILF, SLF, UF, SLF temporal part, medial lemniscus, ICP, SCP, CP, ALIC, PLIC, ACR, SCR, PCR, PTR, sagittal stratum, and external capsule; Lt- retrolenticular part of IC; Rt-CgH; forceps major and minor, MCP, PCT, genu, body, and splenium of CC. | 6.56 | 82, 93, 43 |
| AK | HCs  > PD-nICB | 28345 | Bilateral ATR, CST, CgH, IFOF, ILF, SLF, UF, ALIC, PLIC, retrolenticular part of IC, ACR, SCR, PCR, PTR, sagittal stratum, and SFOF; Lt-CCG and CP; Rt-SLF temporal part and external capsule; forceps major and minor, genu, body, and splenium of CC, and fornix. | 7.88 | 90, 130, 79 |
|  | HCs  > PD-ICB | 19293 | Bilateral ATR, CST, CgH, IFOF, ILF, UF, medial lemniscus, ICP, SCP, CP, ALIC, PLIC, retrolenticular part of IC, ACR, SCR, PCR, sagittal stratum, and SFOF; Lt-CCG; Rt-SLF, SLF temporal part, PTR, and external capsule; forceps major and minor, MCP, PCT, genu, body, and splenium of CC, and fornix. | 6.98 | 90, 130, 79 |
|  | PDs-nICB  < PD-ICB | 6535 | Bilateral ATR, IFOF, ACR, PCR, and PTR; Lt-CST,　ILF, SLF, ALIC, PLIC, retrolenticular part of IC, and SCR; Rt-SFOF; forceps major and minor, genu, body, and splenium of CC. | 4.92 | 54, 153, 88 |
| **NODDI** |  |  |  |  |  |
| ICVF | HCs  > PD-nICB | 23359 | Bilateral ATR, CST, CCG, IFOF, ILF, SLF, UF, SLF temporal part, ALIC, PLIC, retrolenticular part of IC, ACR, SCR, PCR, PTR, sagittal stratum, and external capsule; Lt-SFOF; forceps major and minor, genu, body, and splenium of CC. | 6.27 | 67, 170, 86 |
| ODI | HCs  > PD-nICB | 130 | Rt-IFOF and ACR; forceps minor and genu of CC. | 4.16 | 63, 143, 86 |
|  | HCs  < PD-ICB | 2466 | Lt-ATR, CST, IFOF, ILF, SLF, UF, ALIC, PLIC, retrolenticular part of IC, ACR, SCR, PCR, and PTR; forceps minor, body and splenium of CC. | 4.90 | 132, 153, 85 |
|  | PD-nICB  < PD-ICB | 3694 | Bilateral ATR, CST, IFOF, ALIC, PLIC, retrolenticular of IC, ACR, SCR, PCR, and PTR; Lt-ILF, SLF, UF, and sagittal stratum; Rt- external capsule; forceps minor, body and splenium of CC. | 5.07 | 64, 92, 97 |
| **MT-sat** |  |  |  |  |  |
| MVF | HCs  > PD-nICB | 24974 | Bilateral ATR, CST, IFOF, ILF, SLF, UF, ALIC, PLIC, retrolenticular part of IC, ACR, SCR, PCR, PTR, sagittal stratum, external capsule, and SFOF; Rt-CCG, SLF temporal part, and CP; forceps major and minor, genu, body, and splenium of CC, and fornix. | 6.06 | 123, 117, 67 |
|  | PD-nICB  < PD-ICB | 13251 | Bilateral ATR, CST, IFOF, ILF, SLF, UF, ALIC, PLIC, retrolenticular part of IC, ACR, SCR, PCR, PTR, and sagittal stratum; Lt-SFOF; Rt- SLF temporal part and external capsule; forceps minor, and body and splenium of CC. | 5.65 | 132, 119, 52 |
| Abbreviations: Lt, left; Rt, right; ACR, anterior corona radiata; AD, axial diffusivity; AK, axial kurtosis; ALIC, anterior limb of internal capsule; ATR, anterior thalamic, radiation; CC, corpus callosum, CCG, cingulum cingulate gyrus; CgH, cingulum hippocampus; CP, cerebral peduncle; CST, corticospinal tract; DKI, diffusion kurtosis imaging; DTI, diffusion tensor imaging; FA, fractional anisotropy; HCs, healthy controls; IC, internal capsule; ICBs, impulse control behaviors; ICP, inferior cerebellar peduncle; ICVF, intracellular volume fraction; IFOF, inferior fronto-occipital fasciculus; ILF, inferior longitudinal fasciculus; ISO, isotropic volume fraction; MCP, middle cerebellar peduncle; MD, mean diffusivity; MK, mean kurtosis; MVF, myelin volume fraction; NODDI, neurite orientation dispersion and density imaging; ODI, orientation dispersion index; PCR, posterior corona radiata; PCT, pontine crossing tract; PD, Parkinson’s disease; PD-ICB, Parkinson’s disease patients with impulse control behaviors; PD-nICB, Parkinson’s disease patients without impulse control behaviors; PLIC, posterior limb of internal capsule; PTR, posterior thalamic radiation; RD, radial diffusivity; RK, radial kurtosis; SCP, superior cerebellar peduncle; SCR, superior corona radiata; SFOF, superior fronto-occipital fasciculus; SLF, superior longitudinal fasciculus; TBSS, tract-based spatial statistics; UF, uncinate fasciculus. | | | | | |

**Supplementary Table 3. ROI analysis of the indices with significant differences between PD patients with and without ICBs.**

|  |  | *p*-value^a^ | FDR | HCs | PD-ICB | *p*-value^b^ | PD-nICB | *p*-value^b^ | *p*-value^b^ |  |
| --- | --- | --- | --- | --- | --- | --- | --- | --- | --- | --- |
|  |  |  | *p*-value^c^ | mean (SE) | mean (SE) | (vs HCs) | mean (SE) | (vs HCs) | (vs PD-ICB) |  |
| MD | Fminor | 0.0881 | 0.0899 |  |  |  |  |  |  |  |
|  | GenuCC | 0.0543 | 0.0899 |  |  |  |  |  |  |  |
|  | body of CC | 0.0899 | 0.0899 |  |  |  |  |  |  |  |
|  | splenium of CC | 0.0351 | 0.0899 |  |  |  |  |  |  |  |
|  | ACR | 0.0704 | 0.0899 |  |  |  |  |  |  |  |
| AK | Fminor | 0.104 | 0.1300 |  |  |  |  |  |  |  |
|  | GenuCC | 0.0285 | 0.0560 |  |  |  |  |  |  |  |
|  | BodyCC | 0.0336 | 0.0560 |  |  |  |  |  |  |  |
|  | SpCC | 0.0001 | <0.0001 | 0.770 (0.00924) | 0.753 (0.00948) | 0.0190 | 0.721 (0.00974) | <0.0001 | 0.0298 |  |
|  | SLF | 0.925 | 0.925 |  |  |  |  |  |  |  |
| ODI | leftPLIC | 0.377 | 0.377 |  |  |  |  |  |  |  |
|  | leftRLIC | 0.0262 | 0.0786 | 0.111 (0.00280) | 0.117 (0.00287) | 0.0744 | 0.107 (0.00295) | 0.2482 | 0.0122 |  |
|  | left SLF | 0.0536 | 0.0804 |  |  |  |  |  |  |  |
| MVF | IFOF | 0.0620 | 0.109 |  |  |  |  |  |  |  |
|  | ILF | 0.0237 | 0.0597 | 0.368 (0.00427) | 0.367 (0.00438) | 0.916 | 0.352 (0.00450) | 0.0142 | 0.0199 |  |
|  | SLF | 0.180 | 0.252 |  |  |  |  |  |  |  |
|  | UF | 0.0256 | 0.0597 | 0.311 (0.00482) | 0.308 (0.00495) | 0.641 | 0.293 (0.00509) | 0.0106 | 0.0353 |  |
|  | ALIC | 0.282 | 0.329 |  |  |  |  |  |  |  |
|  | PLIC | 0.463 | 0.463 |  |  |  |  |  |  |  |
|  | EC | 0.0050 | 0.0350 | 0.300 (0.00307) | 0.297 (0.00565) | 0.669 | 0.281 (0.00367) | 0.0024 | 0.0086 |  |
| Abbreviations: ROI, region of interest; PD, Parkinson’s disease; ICBs, impulse control behaviors; PD-ICB, Parkinson’s disease patients with impulse control behaviors; PD-nICB, Parkinson’s disease patients without impulse control behaviors; Lt, left; ACR, anterior corona radiata; AK, axial kurtosis; ALIC, anterior limb of internal capsule; CC, corpus callosum; EC, external capsule; HCs, healthy controls; IFOF, inferior fronto-occipital fasciculus; ILF, inferior longitudinal fasciculus; MVF, myelin volume fraction; ODI, orientation dispersion index; PLIC, posterior limb of internal capsule; RLIC, retrolenticular part of internal capsule; SLF, superior longitudinal fasciculus; UF, uncinate fasciculus.  ^a^*p*-values were obtained by Kruskal–Wallis test. ^b^*p-*values were obtained by Mann–Whitney test. ^c^*p*-values were corrected by FDR, false discovery rate. | | | | | | | | | | |

**Supplementary Table 4. TBSS analysis of ODI and MVF in Parkinson’s disease patients with and without impulsive-compulsive behaviors adjusted for age, sex, and levodopa equivalent daily dose**

| **Modality** | **Contrast** | **Cluster size** | **Anatomical region** | **Peak**  ***T*-value** | **Peak MNI coordinates**  **(X, Y, Z)** |
| --- | --- | --- | --- | --- | --- |
| **NODDI** |  |  |  |  |  |
| ODI | PD-ICB  > PD-nICB | 5232 | Bilateral ATR, CST, IFOF, SLF, ALIC, PLIC, retrolenticular part of IC, ACR, SCR, and PCR; Lt-ILF, UF, and PTR; Rt-external capsule; Forceps minor, genu, body, and splenium of CC | 5.61 | 113, 140, 89 |
| **MT-sat** |  |  |  |  |  |
| MVF | PD-ICB  > PD-nICB | 24359 | Bilateral ATR, CST, IFOF, ILF, SLF, UF, medial lemniscus, SCP, CP, ALIC, PLIC, retrolenticular part of IC, ACR, SCR, PCR, PTR, sagittal stratum, external capsule, fornix, and SFOF; Lt-CHp; Rt-CCG and SLF temporal part; Forceps major and minor, body and splenium of CC, MCP, and pontine crossing tract | 7.09 | 59, 136. 78 |

Lt, left; Rt, right; ACR, anterior corona radiata; ALIC, anterior limb of internal capsule; ATR, anterior thalamic, radiation; CC, corpus callosum, CCG, cingulum cingulate gyrus; CgH, cingulum hippocampus; CP, cerebral peduncle; CST, corticospinal tract; IC, internal capsule; IFOF, inferior fronto-occipital fasciculus; ILF, inferior longitudinal fasciculus; MT-sat, magnetization transfer saturation; PCR, posterior corona radiata; PD-ICB, Parkinson’s disease with impulsive-compulsive behaviors; PD-nICB, Parkinson’s disease without impulsive-compulsive behaviors; PLIC, posterior limb of internal capsule; PTR, posterior thalamic radiation; SCP, superior cerebellar peduncle; SCR, superior corona radiata; SFOF, superior fronto-occipital fasciculus; SLF, superior longitudinal fasciculus; TBSS, tract-based spatial statistics; UF, uncinate fasciculus.

**Supplementary Table 5. Methods and parameters used in this study.**

| Diffusion tensor imaging (DTI) | Fractional anisotrophy (FA) | Overall directionality of water diffusion |
| --- | --- | --- |
|  | Mean diffusivity (MD) | Magnitude of isotropic diffusion |
| Diffision kurtosis imaging (DKI) | Mean kurtosis (MK) | Microstructural complexity or heterogeneity |
|  | Axial kurtosis (AK) | Microstructural complexity or heterogeneity  along the direction of maximal diffusion |
|  | Radial kurtosis (RK) | Microstructural complexity or heterogeneity  perpendicular the direction of maximal diffusion |
| Neurite orientation dispersion and density imaging (NODDI) | intracellular volume fraction (ICVF) | neurite density |
|  | orientation dispersion index (ODI) | Dispersion of neurites in the intracellular compartment |
| magnetization transfer saturation (MT-sat) imaging | myelin volume fraction (MVF) | myelin volume |
